# Polymer-Functionalized Magnetic Nanoparticles for Targeted Quercetin Delivery: A Potential Strategy for Colon Cancer Treatment

**DOI:** 10.3390/pharmaceutics17040467

**Published:** 2025-04-03

**Authors:** Júlia Borges de Macedo, Julia Narayana Schoroeder Bueno, Carla Cristine Kanunfre, José Ricardo de Arruda Miranda, Andris Figueiroa Bakuzis, Priscileila Colerato Ferrari

**Affiliations:** 1 Department of Pharmaceutical Sciences, Ponta Grossa State University (UEPG), Ponta Grossa 84030-900, PR, Brazil; julia.macedo@uepg.br (J.B.d.M.);; 2 Department of General Biology, Ponta Grossa State University (UEPG), Ponta Grossa 84030-900, PR, Brazil; 3 Department of Biophysics and Pharmacology, Institute of Biosciences, São Paulo State University (UNESP), Botucatu 18618-689, SP, Brazil; 4 Institute of Physics, Federal University of Goiás, Goiânia 74690-900, GO, Brazil

**Keywords:** manganese ferrite, cancer therapy, surface functionalization, drug delivery, magnetic drug targeting, folate chitosan, cytotoxicity

## Abstract

**Background/Objectives**: Nanoparticle-based drug delivery systems improve pharmacokinetic aspects, including controlled release and drug targeting, increasing therapeutic efficacy, and reducing toxicity in conventional colon cancer treatment. The superparamagnetism of magnetic nanoparticles (MNP) appears to be a potential alternative for magnetothermal therapy, inducing tumor cell death by an external magnetic field. Therefore, this study aimed to develop chitosan (CS) and folate-chitosan (FA-CS)-coated MNP to improve the stability and targeting of the system for quercetin (Q) delivery. **Methods**: After FA-CS synthesis and 3^2^ factorial design, polymer-functionalized MNPs were produced for quercetin loading, characterized, and evaluated by drug dissolution and cytotoxicity assay. **Results**: The factorial design indicated the positive influence of CS on MNPs’ Zeta potential, followed by the CS–temperature interaction. Optimized formulations had hydrodynamic diameters of 122.32 ± 8.56 nm, Zeta potentials of +30.78 ± 0.8 mV, and loading efficiencies of 80.45% (MNP-CS-Q) and 54.4% (MNP-FA-CS-Q). The 24 h drug release was controlled in MNP-CS-Q (up to 6.4%) and MNP-FA-CS-Q (up to 7.7%) in a simulated tumor medium, with Fickian diffusion release mechanism correlated to the Korsmeyer–Peppas model (R > 0.99). The cytotoxicity assay in HCT-116 showed a higher (*p* < 0.001) dose-dependent antitumor effect of quercetin-loaded MNP compared to free drug, with IC50s of 1.46 (MNP-CS) and 1.30 µg·mL^−1^ (MNP-FA-CS). **Conclusions**: Therefore, this study contributes to the development of biomedical nanotechnology and the magnetic debate by highlighting the antitumor potential of quercetin magnetic nanoparticles. The experimental design allows the discussion of critical manufacturing variables and the determination of optimal parameters for the formulations.

## 1. Introduction

Magnetic nanoparticles (MNP) have attracted enormous attention in biomedical applications, particularly cancer therapy, due to their nanometer size and high surface area. Their superparamagnetic properties enable their use as therapeutic adjuvants, inducing tumor cell death through magnetic hyperthermia and ferroptosis in the case of iron oxide nanoparticles. Furthermore, research over the last few years has shown the benefits of these nanoparticles being controllable by external magnetic fields, which facilitate the delivery of drugs to specific sites in the organism, reduce the systemic distribution of cytotoxic compounds, and increase treatment efficiency [1,2,3,4].

The application of superparamagnetic iron oxide nanoparticles (SPIONs) in drug delivery is well established due to their extensively reported therapeutic, theranostic, and toxicity profiles, with regulatory approval. To further optimize their properties, applications, and production, researchers have explored alternatives involving ferrite nanoparticles, whose characteristics are intrinsically determined by the incorporated cation (MFe_2_O_4_, where M can be Ni, Cu, Zn, Fe, Mn, or Co). Recent studies have investigated the incorporation of Zn^2+^ and Mn^2+^ into iron oxide, leading to enhanced hyperthermia effects with precise temperature control. This advancement minimizes damage to surrounding healthy tissues, highlighting the potential of mixed oxides for cancer therapy [5,6].

However, to maintain these nanomedicine properties, it is necessary to avoid MNP aggregation. Surface coating strategies using polymers represent recent advances in functionalizing MNPs in core–shell structures, ensuring nanoparticles’ physical and chemical stability and high biocompatibility, safety, and improved cell interaction and internalization. In addition, it provides functional groups for conjugation to drugs and targeted ligands [1,2,7]. Chitosan (CS) is an interesting biopolymer with controlled drug release and mucoadhesion properties, which increases the residual time in colonic absorption and provides reactive amine groups after coating. It was further revealed that CS could be conjugated with folic acid (FA), improving specific delivery to the tumor site by the overexpression of the folate receptor in some cancer cells and accelerating controlled cellular uptake [8,9].

Several studies have reported functionalized MNPs as drug delivery systems for cancer therapy by combining the potential cytotoxicity of MNP with chemotherapy and magnetic targeting, including the MNPs used in this study [3,8,10,11]. In a recent work, PEGylated magnetoliposomes of MnFe_2_O_4_ were successfully developed to control doxorubicin in cancer via folate targeting, similar to the approach proposed in this study. However, Cintra et al. demonstrated the hyperthermia-mediated controlled release of the nanoparticle, increasing in vitro cytotoxicity in tumor cells [11]. Nejadshafiee et al. demonstrated the promising results of a chitosan-folate decorated SPION, a bio-metal–organic magnetic nanocomposite for the simultaneous delivery of curcumin and 5-fluorouracil (5FU) with theranostic potential in breast cancer [8]. Dabaghi et al. demonstrated the therapeutic effect of chitosan-coated SPION with 5-FU and magnetic hyperthermia in a heterotopic tumor model of human colorectal cancer (HT29) in mice [10].

Quercetin is a polyphenolic phytochemical with potent antioxidant, anti-inflammatory, and anticancer activities, inducing cell death in colon cancer through various molecular mechanisms. However, due to its limited solubility and stability, nanoencapsulation and targeted delivery strategies are needed to increase the bioavailability and potential of anticancer drugs [12,13,14]. It has been demonstrated that quercetin-loaded CS nanoparticles enhanced the pharmacokinetic properties, enhancing the antiangiogenic and proapoptotic efficacy against colon cancer [15]. Mandić et al. highlighted the potential of mesoporous magnetite nanoparticles (MNPs) for quercetin delivery, achieving sustained and controlled release kinetics by applying combined alternating and steady-state magnetic fields [16].

Nanostructured carrier systems’ design for active compounds represents an attractive targeted therapy strategy that must be carefully engineered to maintain optimal pharmacokinetic and biodistribution characteristics [3]. Despite significant advances, few studies have investigated the use of quercetin-loaded MNPs for colon cancer treatment. Therefore, in this study, FA-CS-functionalized MnFe_2_O_4_-based MNPs were designed to enhance cytotoxic effects through therapeutic synergy and targeted quercetin delivery. This development involves loading quercetin into the polymeric layer, which coats the magnetic core. Physicochemical characterization and MTT cytotoxicity assays in human colon cancer cells were performed to assess the produced nanoparticulate system and provide preliminary insights into its cytotoxic potential.

## 2. Materials and Methods

### 2.1. Materials

We used quercetin (≥95%, Sigma-Aldrich Brazil Ltda., Cajamar, Brazil), chitosan (low molecular weight, Sigma-Aldrich Brazil Ltda.), folic acid (≥97%, Sigma-Aldrich Brazil Ltda.), 1-(3-dimethylaminopropyl)-3-ethylcarbodiimide hydrochloride—EDC (Sigma-Aldrich Brazil Ltda.), acetic acid (Biotec Eireli-me, São Paulo, Brazil), methanol (HPLC grade, Lichrosolv reag. Ph eur Merck, Germany), ethanol (Neon Commercial Ltda., Suzano, Brazil), Dimethyl sulfoxide-DMSO (Neon Commercial Ltda., Brazil), tween80 (Synth, Diadema, Brazil), RPMI 1640 (Sigma-Aldrich Brazil Ltda.), FBS (Vitrocell, Campinas, Brazil), MTT (Sigma-Aldrich Brazil Ltda.), distilled water (model 724/2-a, Fanem Ltda., São Paulo, Brazil), ultrapure water (milli-q^®^, Millipore, Bedford, MA, USA), HCT-116 (ATCC CCL-247) (Banco de Células do Rio de Janeiro—BCRJ, Duque de Caxias, Brazil).

### 2.2. MNP Functionalization

Manganese ferrite-based MNP, surface-coated with citrate, was synthesized using the co-precipitation procedure previously described [17]. The polymer coating solution (25 mL of 2% (*v*/*v*) acetic acid) and 0.25 mL of magnetic fluid (38 mg·mL^−1^ of MNP) were stirred in a shaker incubator (430-RDBP, Ethik Technology, Vargem Grande Paulista, Brazil) at 1500 rpm for 1 h. The functionalized MNPs were collected after 15 days of lateral magnetic separation and suspended in 1 mL of distilled water for 1 min using a probe sonicator (power of 550 watts and frequency in 20 kHz with amplitude of 80%, 2 s on 2 s off—Eco-Sonics, Indaiatuba, Brazil; QR550).

### 2.3. Design of Experiment (DoE)

This study used a 3^2^ factorial design to optimize the functionalization of MNP by two factors: chitosan concentration (0.5, 1, and 2 mg·mL^−1^) and stirring temperature (20, 30, and 40 °C). The experiments were conducted with three levels (−1; 0; +1) of each variable in duplicate, resulting in 18 experiments. To optimize the formulation using the hydrodynamic diameter (Dh) and Zeta potential (ζ) responses, a multiple factorial regression analysis was applied using a quadratic system and polynomial terms:

(1) $$Y=\beta_{0}+\beta_{1}X_{1}+\beta_{2}X_{2}+\beta_{12}X_{12}+\beta_{11}X_{1}^{2}+\beta_{22}X_{2}^{2}$$

where Y is the dependent variable, β is the regression coefficient, 0 is the arithmetic mean response, and 1 and 2 correspond to the CS and temperature factors. In addition, statistical validation was established by analysis of variance (ANOVA) to verify the fitted model and significant influences. Formulation optimization was defined based on composite desirability (d), a global index created by combining each factorial design’s response variables and factors. Following the definition of the best factors for functionalization, the optimized MNP was coated with chitosan and chitosan–folate for quercetin delivery.

### 2.4. Folic Acid–Chitosan Conjugation Synthesis

FA-CS conjugate was synthesized according to a similar procedure reported by [18]. The FA (0.73 g) and EDC (0.69 g) were dissolved in 50 mL of anhydrous DMSO while magnetically stirring at room temperature. FA-EDC solution was slowly dropped into 100 mL of CS solution (0.5% *w*/*v*) in acetic acid (0.1 M) and stirred in the dark for 24 h at room temperature. Finally, NaOH (1 M) was added to increase the pH to 9.0, allowing the conjugate to precipitate. The FA-CS was then collected by centrifugation at 5000 rpm for 15 min, rinsed with excess distilled water, and freeze-dried. The supernatant was measured using a UV spectrophotometer (M51, Bel Engineering, Newcastle upon Tyne, UK) at 285 nm. The method and calibration curve were previously standardized (concentration range of 2–12 µg·mL^−1^; y = 0.05361x − 0.02229; r^2^ = 0.99). The conjugation efficiency (CE) and weight of FA conjugated (FAc) were estimated using the equations below:

(2) $$CE\;\left(\%\right)=\frac{\left(Theoretical\;amount\;of\;Folic\;Acid-Measured\;amount\;of\;Folic\;Acid\right)}{Theoretical\;amount\;of\;Folic\;Acid}\times 100$$

(3) $$FAc\;\left(g\right)=Weight\;of\;theoretical\;Folic\;acid-Weight\;of\;free\;Folic\;acid$$

### 2.5. Quercetin Loading

Quercetin was loaded into CS or FA-CS-functionalized MNP using a shaker incubator (430-RDBP, Ethik Technology, Brazil). Briefly, 10 mg of functionalized MNP were dispersed in 10 mL ethanolic quercetin solution (400 µg·mL^−1^) and were shaken at 1500 rpm for 1 h at room temperature, followed by a 24 h rest to facilitate quercetin uptake. Then, quercetin-loaded MNPs were collected after 15 days of lateral magnetic separation and suspended in 1 mL of distilled water for 1 min using a probe sonicator (amplitude 80% 2 s on 2 s off—Eco-Sonics, QR550). The loading efficiency was quantified by measuring the absorbance values of the unloaded drug in the supernatant with a UV spectrophotometer (M51, Bel Engineering) at 373 nm. The method has previously been validated and standardized (concentration range of 2.0–12.0 µg·mL^−1^ in methanol; y = 0.07468x + 0.00222; r^2^ = 0.99). The percentages of loading efficiency (LE) and drug loading (DL) were calculated by the following equations:

(4) $$LE\;\left(\%\right)=\frac{\left(Quercetin\;initial-Quercetin\;free\right)}{Quercetin\;initial}\times 100$$

(5) $$DL\;\left(\%\right)=\frac{Weight\;of\;the\;loaded\;quercetin}{Weight\;of\;the\;Nanoparticles}\times 100$$

### 2.6. Physicochemical Characterization

The Zetasizer (Nano ZS90, Malvern Instruments, Malvern, UK) was used to measure the hydrodynamic diameter (Dh) and polydispersity index (PdI) using dynamic light scattering (DLS) and Zeta potential (ζ) via laser Doppler microelectrophoresis. The samples were diluted in distilled water (1:200 *v*/*v*), in triplicate, at room temperature, and the data were analyzed using cumulant statistics. The photomicrographs were evaluated using a Tescan Mira3 (Brno-Kohoutovice, Czech Republic) field emission gun scanning electron microscope (FEG-SEM) set to 10 kV with magnifications ranging from 50 to 150 kx. The MNPs (1:500 *v*/*v* in distilled water) were coated with a gold–palladium layer for surface visibility (IC-50 Ion Coater equipment, Shimadzu, Kyoto, Japan), and the average size of 100 particles was measured using ImageJ software (1.52n Java 1.8.0_333). The samples’ Fourier transform infrared (FT-IR) spectra in KBr pellets were recorded using a Shimadzu spectrometer (IR Prestige-21) in the range of 400–4000 cm^−1^, with a resolution of 4 cm^−1^ and 32 scans·min^−1^. Crystallinity patterns were acquired with a Rigaku (Ultima IV) X-ray diffractometer (XRD) using Cu Kα radiation (λ = 0.15418 Å) with a step increment 2θ = 0.02, ranging from 5° to 50° (tube operating at 40 kV/40 mA). The thermal stability of the nanoparticles was evaluated simultaneously using thermogravimetric analysis (TGA) and differential scanning calorimetry (DSC) in a synthetic air atmosphere ranging from 25 to 800 °C, at a rate of 10 °C·min^−1^, using an STA 6000 (Perkin Elmer, Waltham, MA, USA).

### 2.7. In Vitro Drug Release

The quercetin release profile from MNP was investigated according to Shah et al. (2013) with modifications [19]. At a ratio of 1:10 (*v*/*v*), the nanoparticles were added to the simulated tumoral dissolution medium (PBS pH 5.8 with 0.5% tween 80^®^ *v*/*v*) and kept in a shaker incubator (430-RDBP, Ethik Technology, Brazil) at 100 rpm and 37 °C for 24 h. The quercetin quantification was performed by UV spectrophotometry after the centrifugation of the samples (10.000 rpm for 10 min at 4 °C) in an ultracentrifuge (Table Top Refrigerated Centrifuge Z326K, Hermle Labortechnik GmbH, Wehingen, Germany).

The drug release results were fitted to the mathematical models of zero-order, first-order, Higuchi, Korsmeyer–Peppas, and Hixson–Crowell to determine the quercetin release kinetics. The fitting of the experimental data to the model was performed by linearization, and the R was used as the model selection criterion.

### 2.8. Cytotoxicity Assay

The antitumoral activity was evaluated against a human colon cancer epithelial cell line HCT-116 (ATCC CCL-247). The cells were subcultured in RPMI 1640 medium (pH 7.4) supplemented with 10% fetal bovine serum, 24 mmol·L^−1^ sodium bicarbonate, 2 mmol·L^−1^ glutamine, and 10,000 IU penicillin and 10 mg streptomycin, under a humidified atmosphere (5% CO_2_) at 37 °C. These cells were subcultured every 4 days until the end of the experiment.

Cytotoxicity was performed by the MTT [3-(4,5-dimethylthiazol-2-yl)-2,5-diphenyltetrazolium bromide] assay to investigate changes in mitochondrial/non-mitochondrial dehydrogenase activity. The experiment was designed with concentrations of 5 × 10^4^ cells·mL^−1^, seeded in 96-well plates for 24 h under the culture conditions already described. The abilities of MNP-CS, MNP-FA-CS, and loaded and unloaded quercetin to induce HCT-116 cell death were evaluated for 48 h at different concentrations (10–200 μg·mL^−1^) to verify the dose dependence and the IC50, the treatment concentration that inhibited 50% of cell growth, which was calculated by Probit regression (Finney method). To verify the time-dependence relationship, the concentration of 25 μg·mL^−1^ was evaluated for 24, 48, and 72 h of exposure. After the incubation, the cells were rinsed with PBS, followed by adding 100 μL of MTT (0.5 mg·mL^−1^) and incubation for 1 h. Then, the medium was replaced with DMSO (100 µL) to dissolve the formazan crystal formed in each well, and the absorbance (ABS) was measured at 550 nm using a microplate reader (Biotek μQuant, Winooski, VT, USA).

MNPs are dark brown and interfere with spectrophotometry measurements when taken up by cells. The readings were thus adjusted, as Häfeli et al. reported [20], with an absorbance reading containing the same number of cells that had gone through the same washing processes but had not received the MTT solution. The following equation determined the percentage of cell viability (CV):

(6) $$CV\;\left(\%\right)=\frac{\left(ABS\;for\;treated\;cells-ABS\;for\;cells\;treated\;without\;MTT\right)}{ABS\;for\;the\;control\;cells}\times 100$$

### 2.9. Data Analysis

DoE analysis was performed using Minitab^®^ 21.4.1, drug release models were fitted by use of the SigmaPlot^®^ 11.0 software, a cell viability assay was performed with GraphPad Prism^®^ 5.0 and Probit analysis was performed with Stat Plus^®^ 5.8.4. A one-way analysis of variance (ANOVA) with a post-hoc Tukey test was used, and *p*-values < 0.05 were considered statistically significant. The results are presented as mean ± standard deviation from at least three replicate studies.

## 3. Results and Discussion

The MNPs were synthesized with citrate on the surface to add negative charges, facilitating functionalization with chitosan, a cationic polymer. MnFe_2_O_4_–Citrate MNPs were incorporated into chitosan to protect the magnetic core, increase its stability, reduce toxicity, and add functions to the nanocomposite for biomedical applications, such as the ability to load drugs or actively target [21].

In the current study, MnFe_2_O_4_–Citrate MNPs were coated with chitosan for quercetin loading. This system allows passive targeting due to its nanosized size and magnetic targeting when exposed to an external magnetic field directed at the tumor site. Drug targeting minimizes the risk of adverse effects associated with systemic absorption and, in the context of cancer therapy, can reduce nonselective cytotoxicity to healthy cells surrounding the tumor. To further enhance targeting efficiency, folic acid—a specific ligand for tumors overexpressing the folate receptor—was conjugated to chitosan, enabling quercetin loading while coating MnFe_2_O_4_–Citrate. In addition to the advantages of targeting and mucoadhesion, the use of MNPs offers the potential for combination therapy, integrating chemotherapy (via the cytotoxic effects of quercetin) and magnetotherapy (leveraging hyperthermia and ferroptosis induced by iron oxide nanoparticles) to enhance therapeutic efficacy. In this study, we developed two nanocomposite systems, MNP-CS-Q and MNP-FA-CS-Q, and conducted physicochemical characterization analyses along with a preliminary cytotoxicity assay in a colon cancer cell line.

### 3.1. Design of Experiment

The 3^2^ factorial design proposed nine formulations (Table 1), varying the concentration of the polymer coating solution and the processing temperature. Temperature was identified as a critical parameter in the coating process, while polymer concentration was considered a key factor influencing formulation characteristics, particularly particle size (evaluated by hydrodynamic diameter) and surface charge (assessed via Zeta potential). Given their impact on nanoparticle performance, hydrodynamic diameter (Dh) and Zeta potential (ζ) were selected as the primary response variables for evaluation.

Nanoparticles with smaller sizes, typically ranging from 10 to 200 nm, enhance tumor permeability via the enhanced permeability and retention (EPR) effect, allowing for prolonged tumor accumulation while minimizing hepatic clearance. In contrast, Zeta potential provides insight into surface charge characteristics, with values ≥ |30 mV| (in modulus) indicating greater electrostatic stability and reduced susceptibility to agglomeration. Additionally, positively charged nanoparticles may improve drug uptake by cancer cells, further enhancing therapeutic potential [3,22,23].

The design of experiments (DoE) improved the polymeric coating process by varying the components simultaneously and evaluating the responses induced by the independent variables and their interactions (Table 1). As expected, increasing the chitosan (CS) concentration, a positively charged polymer, led to higher Zeta potential values, reaching up to 28.97 mV at the highest concentration (2 mg·mL^−1^). This result indicates good formulation stability and favorable properties for the intended application. However, at lower CS concentrations and reduced temperatures (−, −), the nanoparticles exhibited a negative charge, suggesting poor functionalization. These findings indicate that formulations with 0.5 mg·mL^−1^ of chitosan as the coating solution are not recommended.

The hydrodynamic diameter (Dh) results are particularly intriguing. Contrary to expectations, increasing the CS concentration (+, +/0/−) did not lead to larger particles. This phenomenon may be attributed to an effective coating process, enhancing stability and reducing aggregation. It is important to note that the dynamic light scattering (DLS) technique, used to determine Dh, has limitations as it analyzes nanoparticles in aqueous suspension, measuring both aggregates and the solvation layer surrounding the particles. Consequently, DLS often estimates larger particle sizes than those observed via field-emission gun scanning electron microscopy (FEG-SEM) [24,25].

**Figure 1:**
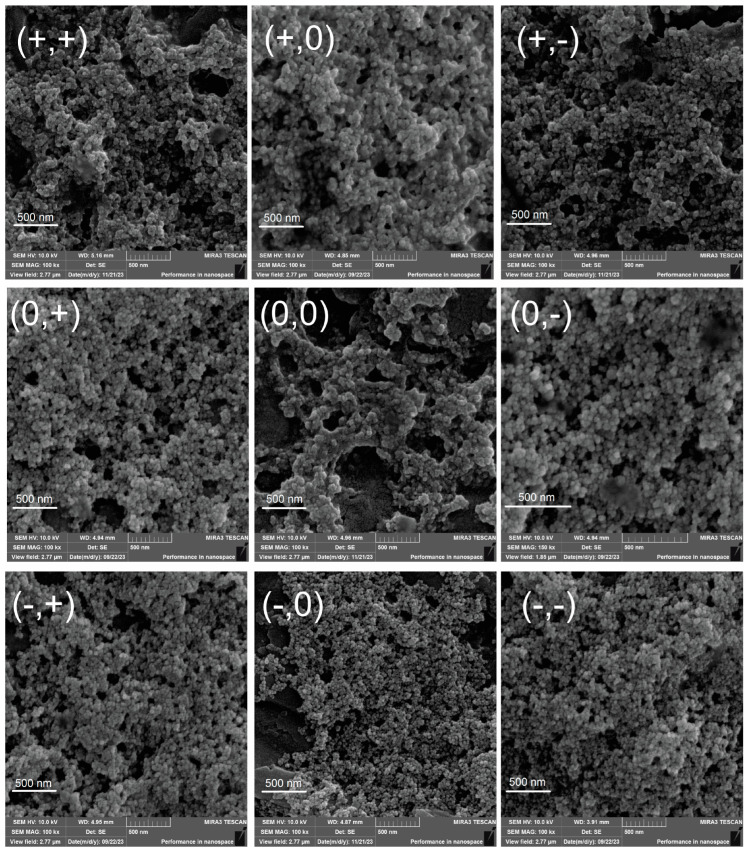
FEG-SEM micrographs of the factorial design formulations.

FEG-SEM provided a more accurate analysis of nanoparticle size in a dry state (Figure 1) without the interference of the solvation shell. In this case, size variations were slight between the additions of CS. In general, nanoparticles tended to be larger when coated with higher polymer concentrations and processed at lower temperatures. Similar findings were reported by Yarjanli et al. (2019), who observed that iron oxide nanoparticles containing quercetin appeared smaller in SEM analysis compared to DLS measurements [26]. In addition, this technique allows for observing the morphology of the nanoparticles and their shape and agglomeration tendencies. All micrographs showed round nanoparticles, with their nanometric sizes quantified and described in Table 1.

**Figure 2:**
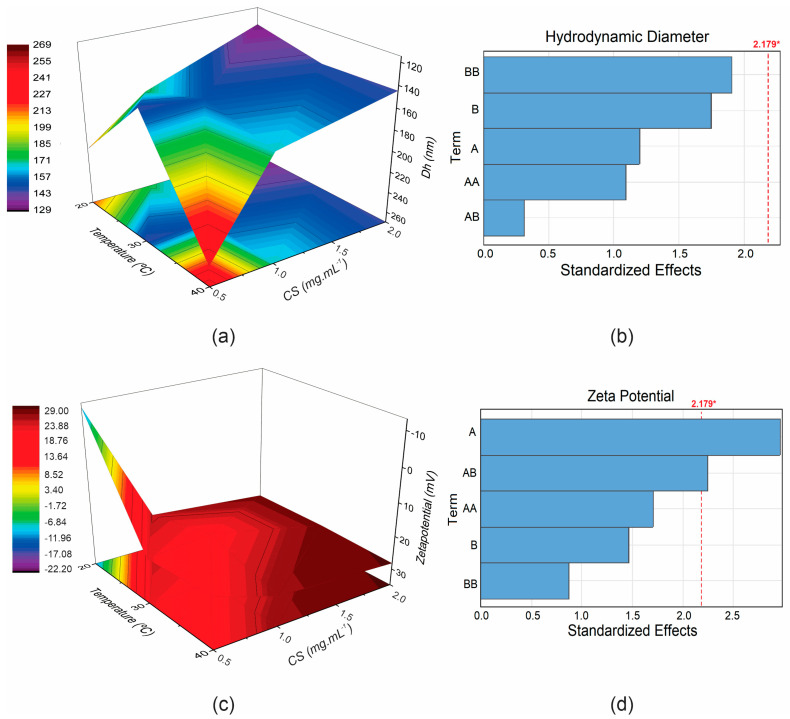
Response surface plots and Pareto chart showing the influence of chitosan concentration and temperature on hydrodynamic diameter (**a**,**b**) and ζ potential (**c**,**d**). Term A: chitosan; B: temperature; * *p* < 0.05.

The results of the factorial analysis are shown in Table 2. The coefficients of the equation indicate the direct (positive value) or inverse (negative value) influence of the factors on the response. The quadratic linear regression model was validated only for the Zeta potential effect (R^2^ of 0.6757), with a significant influence (*p* < 0.05) for CS and CS-°C interaction. This influence is evident in the 3D response surface plots (Figure 2), which allow for the analysis of both the individual effects of the variables and their interactions. Additionally, the Pareto diagrams (Figure 2) estimate the magnitude and significance of each variable’s effect on the Dh and ζ of the nanoparticles.

The interaction between chitosan concentration and temperature during the MNP coating process, as observed in the hydrodynamic diameter response (Figure 2a), can be more clearly visualized in the response surface plot. Different temperatures resulted in distinct particle sizes, mainly when the chitosan concentration was lower (0.5 mg·mL^−1^). Intermediate Dh values were observed at 20 °C, while larger nanoparticles were formed at 40 °C. Notably, 30 °C appeared to be a critical temperature, resulting in the lowest Dh values at all chitosan concentrations. Similarly, the influence on Zeta potential can be seen in Figure 2c, with a more pronounced effect from the polymer concentration. As chitosan concentration increases, the particle’s positive charge also increases, indicating successful coating. This trend was also observed with higher temperatures (30 °C and 40 °C), particularly at intermediate and high chitosan concentrations. Poor Zeta potential results were observed at 20 °C with 0.5 mg·mL^−1^ of chitosan, suggesting inadequate functionalization under these conditions.

In the Pareto diagram (Figure 2b,d), the length of each bar represents the standardized effect of the variable or its interaction with the responses. Term A refers to chitosan, B refers to temperature, and AB denotes the interaction between the two. The factor analysis was conducted using a quadratic model, and as such, the interaction terms shown in the diagram include AA (quadratic effect of chitosan) and BB (quadratic effect of temperature). In addition, the diagram includes a dashed line representing the threshold for significant influence (*p* < 0.05), as summarized in Table 2. Bars extending beyond this dashed line indicate statistical significance. Notably, in Figure 2d, the bar corresponding to chitosan and the chitosan-temperature interaction exceeds the dashed line, highlighting its significant influence on the zeta potential of the nanoparticles, with chitosan showing the most significant impact.

In summary, the CS variable was the most critical parameter; higher amounts resulted in smaller diameters and higher Zeta potentials. The medium-level temperature (30 °C) was suitable for smaller diameters and higher zeta potentials, regardless of the CS concentration. The response optimization determines the best level of the parameters (chitosan and temperature) for maximal Zeta potential optimization and minimal hydrodynamic diameter based on the composite desirability (d) calculation [27]. The optimal solution (d = 1) was found at 1.71 mg·mL^−1^ of CS and 29.29 °C (Dh = 118.14 nm; ζ = 32.07 mV). This analysis suggests the production of MNP using 2 mg·mL^−1^ of CS at 30 °C, as seen in the response surface graph.

### 3.2. Folic Acid-Chitosan Conjugation

**Figure 3:**
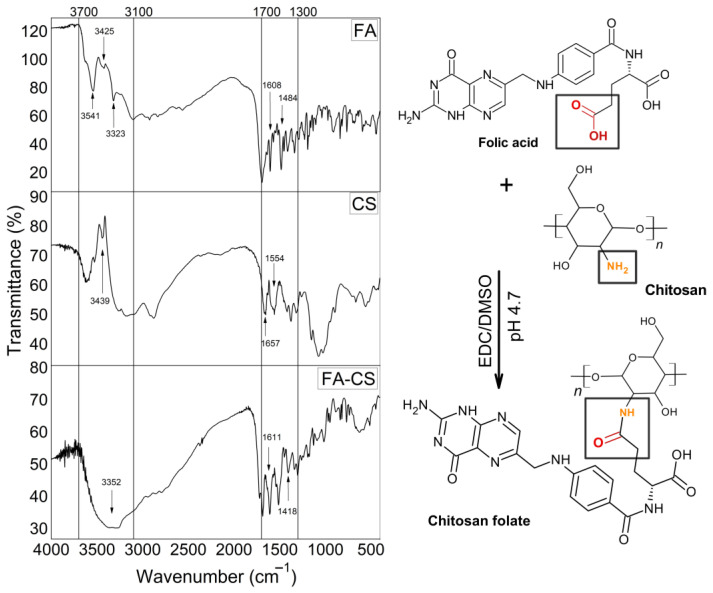
Chitosan folate conjugation reaction and FT-IR spectra for characterizing conjugated.

The conjugation of the carboxylic group of folic acid (R-COOH) and the amine of chitosan (R-NH_2_) is promoted by the carbodiimide reaction in the presence of the coupling agent EDC (Figure 3). This chemical interaction resulting in chitosan folate was validated by FT-IR analysis and the conjugation efficiency with the amount of folic acid [18,28].

The chitosan folate was characterized (Figure 3) by the appearance of a broad band at 3352 cm^−1^ due to the overlap of R-OH (3541 cm^−1^), NH of secondary amide (3323 cm^−1^) and primary amine (3425 cm^−1^) of folic acid and the OH vibration of chitosan (3439 cm^−1^). The disappearance of the secondary amine CS band at 1554 cm^−1^ and the shift of the primary amide from 1657 cm^−1^ to 1611 cm^−1^ also showed the CO-NH bond between folic acid and chitosan, indicating a conjugation [28,29,30,31]. The FA spectrum also showed characteristic bands at 1695, 1608, and 1484 cm^−1^, indicating the C=O bond (which forms a new CO-NH bond in FA-CS), amino group on the pteridine ring and C=C or C=N, respectively. The band at 1418 cm^−1^ indicated the elongation of the pteridine ring of FA into conjugated FA-CS [28,31]. Furthermore, the observed N–H bending band at 1517 cm^−1^ is specific to the secondary amide group, which supports the successful conjugation reaction [29,30]. FA-CS was successfully synthesized, containing 0.532 g of FAc (SD ± 0.14) with a CE of 72.92% (SD ± 0.29), an efficiency similar to the results reported by Yang and collaborators [32].

### 3.3. Optimized Nanoparticles

The MNPs optimized by 3^2^ factor analysis were synthesized and characterized (Table 3). Polymer functionalization was confirmed by the inversion of the negative surface charge of MNP (−23.93 mV) to positive in MNP-CS and MNP-FA-CS (+30.78 and 48.38 mV) and an increase in diameter, with a significant difference by ANOVA and Tukey’s post-hoc analysis. The same can be observed when quercetin is loaded (MNP-CS-Q and MNP-FA-CS-Q), changing the surface charge to negative (referring to Q) with an increase in diameter. However, statistically, the mean hydrodynamic diameter of the quercetin delivery system did not differ between non-conjugated-chitosan (MNP-CS-Q) and conjugated (MNP-FA-CS-Q). There are changes in the zeta potential and quercetin loading (LE and DL) when comparing CS to FA-CS. The addition of chitosan-folate (MNP-FA-CS) had a higher surface charge than the addition of non-conjugated CS (MNP-CS), which could be attributed to better interaction between the folate (negative charge) and the iron molecule (positive charge) in the manganese ferrite nanoparticle (MnFe_2_O_4_). In this case, the exposed chitosan component may be responsible for the particle’s higher positive surface charge.

**Figure 4:**
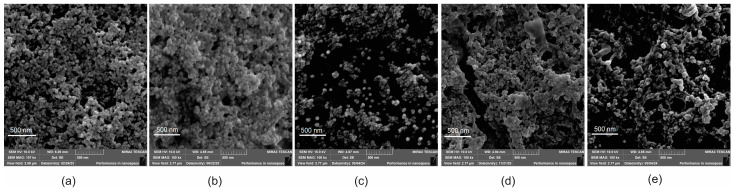
FEG-SEM micrographs of optimized nanoparticles. MNP (**a**); MNP-CS (**b**); MNP-FA-CS (**c**); MNP-CS-Q (**d**); MNP-FA-CS-Q (**e**).

Concerning LE and DL, the loading of quercetin was better with non-conjugated CS and may be related to the thicker polymer layer (greater amount of free CS compared to FA-CS) and also the interaction between quercetin (negative charge) and the magnetic core (positive charge). In micrographs (Figure 4), the size variation was minimal between the samples and smaller than the DLS measurement, following the same theories described in DoE. The MNPs had a spherical morphology (ranging from 20 to 60 nm), and a tendency to agglomerate is noted, as is related to factorial design. The presence of quercetin did not cause significant changes in the micrographs.

The coating, morphology, size, and surface charge of magnetic nanoparticles developed for combined therapy of magnetic hyperthermia and chemotherapy are crucial for both the biodistribution and clearance process and the therapeutic effects, increasing the efficacy of hyperthermia and chemotherapy with greater cellular uptake. Several studies have reported this relationship [1,8,10,19,26,29]. For example, Dabaghi et al. developed iron oxide nanoparticles coated with non-conjugated CS for the delivery of 5-FU, reporting a particle diameter of 98 nm without the drug and 179 nm after drug loading, with Zeta potential values of +20.1 mV and −27.8 mV, respectively. This study demonstrated a greater therapeutic effect when MNPs were combined with 5-FU compared to their use alone in an in vivo model of xenografted human colon tumors (HT-29) in mice. In some cases, there was complete elimination of the tumor when hyperthermia was combined with chemotherapy [10], reinforcing the importance of optimizing particle size and maintaining Zeta potential values close to the electrostatic stability threshold (≥|30| mV) to ensure the biomedical application of nanoparticles.

### 3.4. Physicochemical Characterization

**Figure 5:**
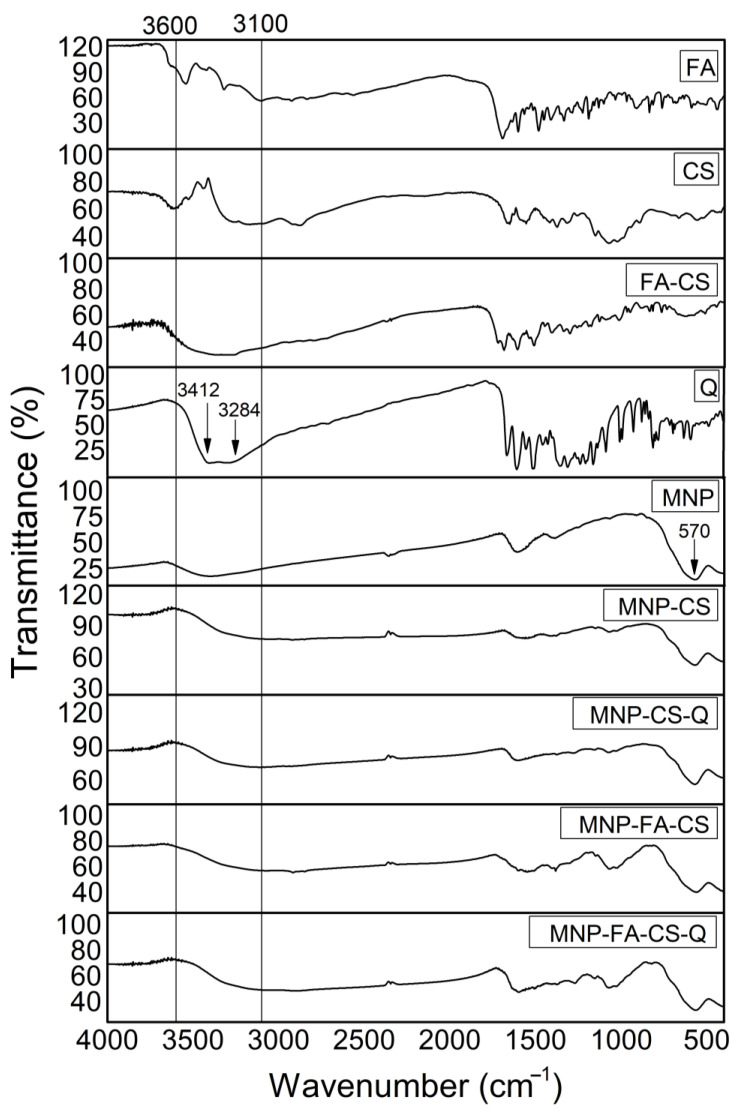
Fourier-transform infrared spectroscopy (FT-IR) spectra of 4000–400 cm^−1^.

The chitosan coating on MNP and the incorporation of quercetin were confirmed and characterized by FT-IR, XRD, and DSC/TGA measurements. In FT-IR (Figure 5), chitosan exhibits characteristic bands at 1657 cm^−1^ corresponding to amide I (C=O stretching) vibrations and 1558 cm^−1^ for amide II (NH in-plane deformation) groups [33]. The band at 570 cm^−1^ is assigned to the Fe-O and Mn-O stretching vibration mode, whereas the bands at 1608 and 3417 cm^−1^ (O-H) are attributed to adsorbed water [34]. The quercetin spectrum shows bands at 819 cm^−1^ (C-O), 3412 and 3284 cm^−1^ (O-H), 1674 cm^−1^ (C=O of carbonyl compounds), and 1620 and 1510 cm^−1^ (C-H). The incorporation into nanoparticles causes the elimination of distinctive quercetin bands, as Barreto et al. (2011) reported [35]. The similarity of the MNP, MNP-CS, MNP-FA-CS, MNP-CS-Q, and MNP-FA-CS-Q spectra indicates structural stability and quercetin, CS, and FA-CS adsorption on the magnetic nanoparticle with no chemical interactions.

**Figure 6:**
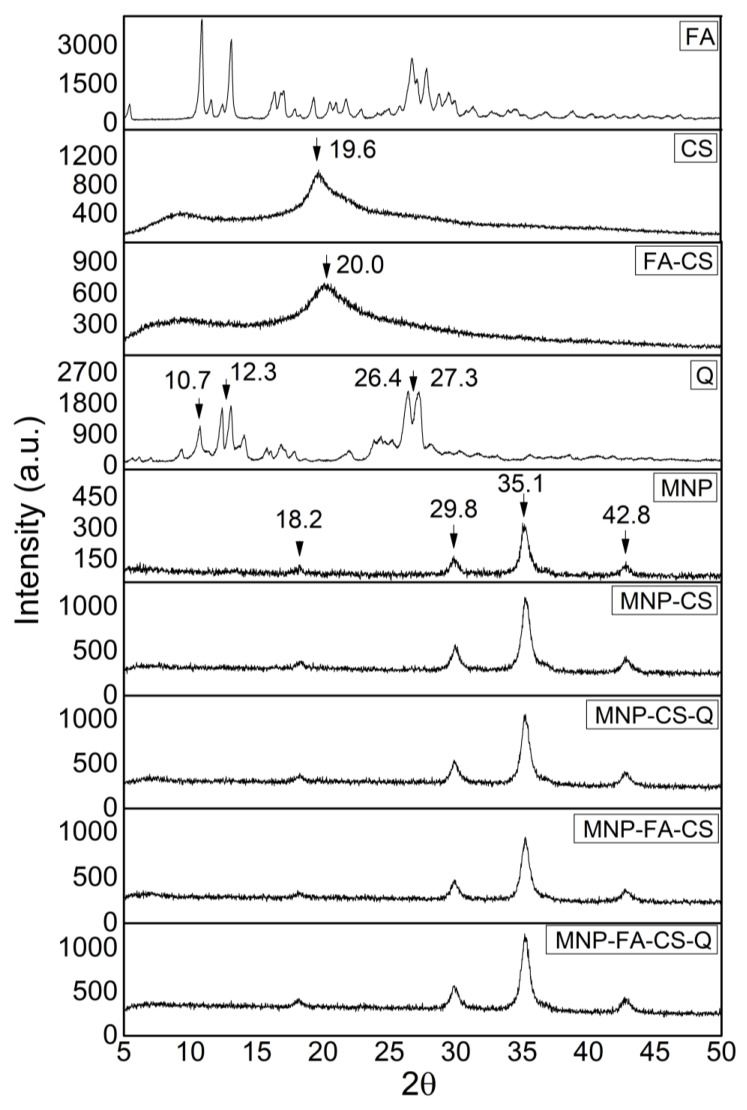
X-ray diffractogram patterns of the nanoparticles.

XRD patterns were obtained to characterize the structure and are shown in Figure 6. The characteristic peaks of MNFe_2_O_4_ at 18.2° (111), 29.8° (220), 35.1° (311), and 42.8° (400) were referred to as crystalline phases following JCPDS 25-0283 [34]. The incorporation of polymers (CS or FA-CS) and quercetin into MNP (MNP-CS, MNP-FA-CS, MNP-CS-Q, MNP-FA-CS-Q) resulted in an intensified crystallographic profile of MnFe_2_O_4_ peaks, suggesting that CS, FA-CS, and quercetin are organized in an amorphous phase. CS and FA-CS showed semi-crystalline characteristics, with broad peaks at 19.6° and 20.0°, respectively [36], while quercetin showed more intense peaks at 10.7°, 12.3°, 26.4° and 27.3°, indicating crystallinity [37].

**Figure 7:**
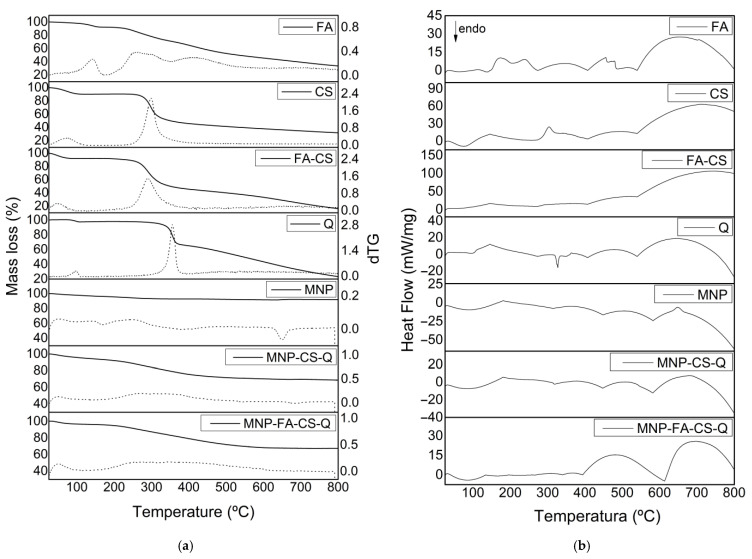
(**a**) Thermogravimetric analysis curves (TG in solid line and dTG in dotted line) and (**b**) Differential scanning calorimetry (DSC).

A thermogravimetric analysis was conducted to understand better the physical and chemical changes that may have occurred during polymer coating and quercetin loading. The thermogravimetric curves (% weight loss) and their derivative (dTG) of MNP with and without CS-Q and FA-CS-Q are shown in Figure 7a. Manganese ferrite exhibits thermal stability [38], and the 5% mass loss at 800 °C may be due to impurities in the sample. At temperatures up to 150 °C, all samples lost mass associated with water removal. Other authors have reported that total degradation of CS, FA-CS, and quercetin occurred at 500 °C. By studying the MNP-CS-Q and MNP-FA-CS-Q thermograms, we may deduce that the 30% mass loss is due to the chemicals incorporated into the MNP [8,31,38].

Analyzing the DSC (Figure 7b), the first thermal event in the compounds can be attributed to water loss. The glass transition of CS has a wide range of variation, between −23 °C and 220 °C, due to its semi-crystalline nature, evidenced in the second endothermic event. The exothermic peak begins at 325 °C, indicates degradation, and shows the thermal stability limit [39,40]. Quercetin’s endothermic peak at 305 °C can be related to its melting point, at a temperature close to that found by [37,41]. The exothermic peak, which begins at approximately 360 °C, reveals its degradation [42]. MNPs do not have thermal events due to their stability at high temperatures. When MNPs are evaluated with CS, FA-CS, and quercetin, it is observed that the degradation and melting point of the drug is suppressed, demonstrating the ability of MNPs to control flavonoid degradation, converting quercetin to an amorphous state.

### 3.5. In Vitro Drug Release

**Figure 8:**
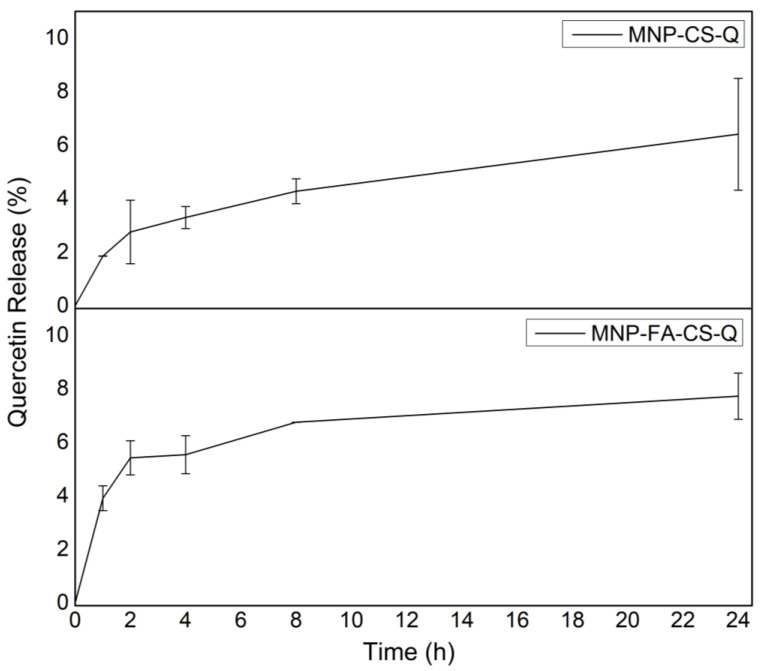
In vitro quercetin release profile of MNP-CS-Q and MNP-FA-CS-Q.

In a dissolution medium similar to the acidic tumor microenvironment (pH 5.6), the release profiles of quercetin-loaded into the surface of coated MNPs (MNP-FA-CS and MNP-CS) are shown in Figure 8. Both showed similar slow-release profiles in medium, with maximum release in 24 h of 7.7% (SD ± 0.86) and 6.4% (SD ± 2.54), respectively, without much difference between the use of conjugated and non-conjugated chitosan. The subtle difference between the systems may be related to more CS when added in free form rather than conjugated (FA-CS). These results corroborate Barreto (2011), who obtained a sustained release of 96 h with a release of only 14.5% of quercetin from polymer-coated MNPs [35]. In a more recent study, Yarjanli et al., conjugated quercetin to iron oxide nanoparticles but without a polymer coating and evaluated a 10-day release profile at a similar acidic pH (pH 4.8), with 58.3% quercetin release on the third day [26]. It is, therefore, possible to conclude that there was a strong interaction between the magnetic core, the coating polymer, and quercetin, making it enjoyable to increase the time of the release assay. Furthermore, superparamagnetic nanoparticles are interesting systems for controlling the release of drugs based on the property of magnetic hyperthermia as a combination therapy. Although not evaluated in this study, magnetic hyperthermia could potentially modulate the quercetin release by applying an external magnetic field.

In addition, the release kinetics using mathematical models was evaluated. According to the kinetic models applied to the release profiles (Table 4), it was possible to demonstrate that the quercetin release from both MNP samples (MNP-FA-CS and MNP-CS) follow the Korsmeyer-Peppas model (R = 0.9924 and 0.9989, respectively). The Korsmeyer-Peppas semi-empirical model represents the fraction of drug released at time t, and n is the release exponent, a parameter that depends on the release mechanism and is thus used to characterize it. The drug transport from MNPs occurs exclusively for Fickian Diffusion (diffusion-controlled release), in which the release exponent was n < 0.4 (MNP-FA-CS: n = 0.1865 and MNP-CS: n = 0.3657). Fickian diffusional release occurs by the usual molecular diffusion of the drug due to a chemical potential gradient [43].

### 3.6. Colon Cancer Cytotoxicity

**Figure 9:**
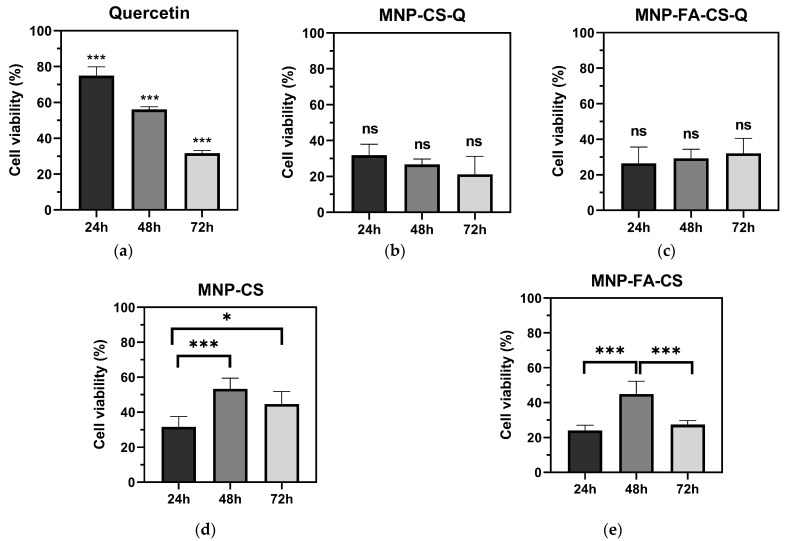
Cell viability analysis by MTT assay: (**a**) quercetin; (**b**) MNP-CS-Q; (**c**) MNP-FA-CS-Q; (**d**) MNP-CS; (**e**) MNP-FA-CS. * *p* < 0.05; *** *p* < 0.0001; ns, no significance.

The MTT assay analyzed the formulations’ cytotoxicity on human colon cancer cells (Figure 9). MNP-CS, MNP-FA-CS, MNP-CS-Q, MNP-FS-CS-Q, and free quercetin were tested for cytotoxicity after 48 h (10–200 µg·mL^−1^). All treatments had a significant concentration-dependent effect on HCT-116 cell viability (CV) when compared to the control (*p* < 0.001). The cytotoxicity of quercetin in these cells has been confirmed with an IC50 of 42.22 µg·mL^−1^. Quercetin loaded into MNP-CS and MNP-FA-CS increased its effect (*p* < 0.0001), with IC50s < 10 µg·mL^−1^ (1.46 and 1.30 µg·mL^−1^, respectively). Even without drug incorporation, functionalized MNPs demonstrated activity, with no significant difference in IC50 values between MNP-CS (19.63 µg·mL^−1^) and MNP-FA-CS (7.75 µg·mL^−1^). This could have influenced the decrease in the IC50 of loaded quercetin, which has more chemotherapeutic potential than free quercetin. On the other hand, the difference in coating polymer, unconjugated CS or FA-CS, showed no significant difference in IC50 between the formulations with or without the drug (MNP-CS vs. MNP-FA-CS and MNP-CS-Q vs MNP- FA-CS-Q). These findings are consistent with a study by Ferrentino et al. (2023) that showed quercetin loaded in polymeric nanoparticles (PCL-PEG-PCL) to be more efficient, with an IC50 of 3.7 µM (1.12 µg·mL^−1^) for the same time and cell line [44].

All treatments were tested at 25 µg·mL^−1^ doses for 24, 48, and 72 h to evaluate the time-dependent relationship. Free quercetin demonstrated a significant direct cytotoxicity effect that increased with exposure time, with CV values of 74.98% (24 h), 56.10% (48 h), and 31.73% (72 h). Quercetin-loaded MNP had lower CVs than free quercetin (range from 21.18 to 32.14%), but there was no significant difference between 24, 48, and 72 h. When evaluated concerning time, this difference in response between free and loaded quercetin may be related to the controlled release of the nanoparticles since they showed a slow release up to 24 h, as discussed above. The functionalized MNPs had a larger effect after 24 h of exposure, with CVs of 31.71% (MNP-CS) and 24.11% (MNP-FA-CS), respectively. In contrast to the formulations used in this investigation, free quercetin has a time-dependent action. However, cell viability was lower when colon cancer cells were treated with the MNPs herein developed.

Although a significant proportion of quercetin remains unreleased in the first 24 h (Figure 8), unloaded MNPs exhibit intrinsic cytotoxicity. Magnetic nanoparticles are not merely passive drug carriers, but have an effect in combination therapy by inducing ferroptosis—a form of iron-dependent cell death mediated by the Fenton reaction, the generation of reactive oxygen species (ROS), and oxidative stress. Furthermore, magnetic hyperthermia may further enhance therapeutic efficacy [2,3,4]. This initial MTT cytotoxicity assay using a single cell line highlights the need for additional studies to elucidate these effects fully. Although minimizing the intrinsic cytotoxicity of MNPs is crucial to optimizing drug efficacy, magnetic targeting can reduce toxicity in healthy cells while enhancing therapeutic effects in tumor cells, thereby minimizing systemic side effects. These aspects should be further explored in future research.

## 4. Conclusions

In this study, we created a novel drug delivery system combining manganese ferrite nanoparticles, chitosan, folic acid, and quercetin, showing favorable morphological and physicochemical properties for targeted therapy with the controlled release of bioactive compounds. The optimization process using factorial design revealed the influence of polymer and temperature in the functionalization, and optimized the conditions to synthesize, nanoparticles with a reduced hydrodynamic diameter (122.32 nm) and colloidal stability. In addition, thermal analyses indicated a possible conversion of quercetin into an amorphous state, which may influence its bioavailability and pharmacokinetic properties. Combining manganese ferrite nanoparticles with quercetin gives rise to a synergism of antitumor activities. Polymer-functionalized magnetic nanoparticles (unloaded) exhibited dose-dependent cytotoxicity (IC50 of 19.63 and 7.75 µg·mL^−1^). The quercetin-loaded MNP showed slow-release control in an acidic dissolution medium (pH 5.6) and increased cytotoxicity (dose-dependent, IC50 < 10 µg·mL^−1^) compared to free quercetin (IC50 of 42.22 µg·mL^−1^), likely due to the synergistic effects between MNP and quercetin. These findings suggest that this delivery system improved quercetin’s efficacy in inhibiting colon cancer proliferation in vitro. Despite these advances, future studies should investigate the mechanisms of cytotoxicity in other human cancer cell lines, and conduct in vivo assays to assess its safety and validate its potential for clinical applications.

## Figures and Tables

**Table 1:** 3^2^ Factorial design parameters and characterization of MNP-CSs.

CS (mg·mL^−1^)	°C	Dh (nm)	PdI *	ζ (mV)	FEG-SEM (nm) *
0.5 (−)	20 (−)	161.43 ± 8.28	0.38 ± 0.03	−1.44 ± 9.75	37.54 ± 8.36
1 (0)	20 (−)	169.10 ± 4.95	0.35 ± 0.01	25.60 ± 0.26	46.29 ± 9.66
2 (+)	20 (−)	133.20 ± 2.01	0.31 ± 0.02	26.63 ± 0.74	31.45 ± 8.78
0.5 (−)	30 (0)	150.13 ± 2.11	0.28 ± 0.05	20.87 ± 1.08	24.46 ± 9.60
1 (0)	30 (0)	157.67 ± 1.78	0.28 ± 0.01	17.43 ± 0.51	34.21 ± 11.52
2 (+)	30 (0)	138.50 ± 2.10	0.38 ± 0.03	26.57 ± 0.55	30.09 ± 9.73
0.5 (−)	40 (+)	235.40 ± 6.22	0.40 ± 0.04	21.93 ± 0.06	32.57 ± 6.53
1 (0)	40 (+)	165.97 ± 3.10	0.38 ± 0.01	27.53 ± 0.76	38.75 ± 21.74
2 (+)	40 (+)	142.13 ± 1.36	0.35 ± 0.01	28.07 ± 1.27	36.15 ± 10.82
0.5 (−)	20 (−)	268.70 ± 91.75	0.39 ± 0.05	−22.17 ± 4.72	-
1 (0)	20 (−)	176.87 ± 4.90	0.32 ± 0.01	23.40 ± 0.62	-
2 (+)	20 (−)	129.40 ± 2.29	0.36 ± 0.01	28.97 ± 1.16	-
0.5 (−)	30 (0)	141.77 ± 0.46	0.26 ± 0.01	22.13 ± 0.85	-
1 (0)	30 (0)	146.33 ± 1.46	0.27 ± 0.01	21.47 ± 0.80	-
2 (+)	30 (0)	154.07 ± 1.96	0.39 ± 0.01	28.53 ± 0.23	-
0.5 (−)	40 (+)	265.77 ± 6.37	0.27 ± 0.05	17.43 ± 0.42	-
1 (0)	40 (+)	168.53 ± 3.65	0.39 ± 0.05	24.40 ± 1.23	-
2 (+)	40 (+)	147.23 ± 3.16	0.40 ± 0.04	28.33 ± 0.15	-

The three levels of DoE were described: (−) lower level, (0) intermediate level, and (+) higher level of each variable; * PdI and FEG-SEM results were not considered in the DoE analysis.

**Table 2:** Summary results of factorial regression.

Terms	β_1_	β_2_	β_12_	β_11_	β_22_	β_0_	R^2^
**Dh**	−121.000 ^ns^	−18,000 ^ns^	−0.500 ^ns^	37.500 ^ns^	0.322 ^ns^	492.000 *	0.556 ^ns^
ζ *	76.400 *	3870 ^ns^	−0.896 *	−0.038 ^ns^	−14.980 ^ns^	−109.600 *	0.676 *

β_0_, constant; β_1_, chitosan; β_2_, time; β_12_, interaction between chitosan and time; β_11_, quadratic interaction of chitosan; β_22_, quadratic interaction of time; R^2^, linearity of the model; * significant influence (*p* < 0.05); ^ns^, non-significant influence.

**Table 3:** Characterization of optimized formulations.

Samples	ζ (mV)	Dh (nm)	PdI	LE (%)	DL (%)
MNP	−23.93 ± 1.45 ^C^	61.38 ± 7.0 ^D^	0.27 ± 0.06 ^C^	-	-
MNP-CS	30.78 ± 0.80 ^B^	122.32 ± 8.56 ^B^	0.46 ± 0.05 ^B^	-	-
MNP-CS-Q	−11.45 ± 1.10 ^D^	388.45 ± 14.50 ^A^	0.69 ± 0.04 ^A^	80.45 ± 4.10 ^A^	24.30 ± 0.93 ^A^
MNP-FA-CS	48.38 ± 4.10 ^A^	93.50 ± 3.98 ^C^	0.23 ± 0.04 ^D^	-	-
MNP-FA-CS-Q	−25.20 ± 1.90 ^E^	365.10 ± 29.01 ^A^	0.38 ± 0.05 ^B^	54.40 ± 5.13 ^B^	17.85 ± 1.40 ^B^

Averages that share the same letter are not significantly different. ANOVA, post-Hoc Tukey; *p* < 0.05.

**Table 4:** Fitting data for in vitro quercetin release profile from MNPs.

Model	Equation	MNP-CS		MNP-FA-CS		Drug Transport Mechanism
		Linear Equation	R	Linear Equation	R	
Zero-order	Q = Q_0_ + K_0_t	y = 0.1779x + 2.3376	0.9320	y = 0.1327x + 4.8285	0.7519	-
First-order	dC/dT = −Kt	y = −0.0008x + 1.9898	0.9356	y = −0.0006x + 1.9785	0.7572	-
Higuchi	Q = KH t^1/2^	y = 0.8817x − 0.8562	0.9896	y = 0.9963x − 3.4131	0.8710	-
Korsmeyer–Peppas	Mt/M∞ = Kt^n^	y = 0.3657x + 2.0074	0.9989	y = 0.1865x + 4.3682	0.9924	Fickian diffusion

Zero order: Q is the amount of drug released or dissolved, Q_0_ is the initial amount of drug in solution, t is time, K_0_ is the zero-order release constant; First order: dC is the concentration derivative, dT is the time derivative, K is the first order rate constant; Higuchi: KH is the Higuchi dissolution constant; Kors-Peppas: Mt/M∞ is the fraction of drug released at time t, K is the rate constant incorporating structural and geometric characteristics of the delivery system, n is the release exponent indicative of the drug’s transport mechanism through the polymer.

## Data Availability

All data will be available via request.

## Abbreviations

MNP	Magnetic nanoparticle
CS	Chitosan
FA-CS	Chitosan folate
Q	Quercetin
Dh	Hydrodynamic diameter
DLS	Dynamic light scattering
PdI	Polydispersity index
PBS	Phosphate buffer solution
HCT-116	Human colon cancer epithelial cell line
IC50	Inhibitory concentration 50%
DSC	Differential scanning calorimetry
TGA	Thermogravimetric analysis
XRD	X-ray diffraction
FT-IR	Fourier-transform infrared spectroscopy
FEG-SEM	Field emission gun scanning electron microscope
